# Insight into genomic organization of pathogenic coronaviruses, SARS-CoV-2: Implication for emergence of new variants, laboratory diagnosis and treatment options

**DOI:** 10.3389/fmmed.2022.917201

**Published:** 2022-10-17

**Authors:** Fikru B. Bedada, Gezahegn Gorfu, Shaolei Teng, Marguerite E. Neita

**Affiliations:** ^1^ Department of Clinical Laboratory Science, College of Nursing and Allied Health Sciences, Howard University, Washington, DC, United States; ^2^ Department of Pathology, College of Medicine, Howard University, Washington, DC, United States; ^3^ Department of Biology, College of Arts and Sciences, Howard University, Washington, DC, United States

**Keywords:** SARS-CoV-2, new genetic variants, genome organization and evolution, molecular diagnosis, ACE2 receptors and COVID-19, vaccines and antiviral drugs, omicron (B.1.1.529), computational model

## Abstract

SARS-CoV-2 is a novel zoonotic positive-sense RNA virus (ssRNA+) belonging to the genus beta coronaviruses (CoVs) in the Coronaviridae family. It is the causative agent for the outbreak of the disease, COVID-19. It is the third CoV causing pneumonia around the world in the past 2 decades. To date, it has caused significant deaths worldwide. Notably, the emergence of new genetic variants conferring efficient transmission and immune evasion remained a challenge, despite the reduction in the number of death cases, owing to effective vaccination regimen (boosting) and safety protocols. Thus, information harnessed from SARS-CoV-2 genomic organization is indispensable for seeking laboratory diagnosis and treatment options. Here in, we review previously circulating variants of SARS-CoV-2 designated variant of concern (VOC) including the Alpha (United Kingdom), Beta (South Africa), Gamma (Brazil), Delta (India), and recently circulating VOC, Omicron (South Africa) and its divergent subvariants (BA.1, BA.2, BA.3, BA.2.12.1, BA.4 and BA.5) with BA.5 currently becoming dominant and prolonging the COVID pandemic. In addition, we address the role of computational models for mutagenesis analysis which can predict important residues that contribute to transmissibility, virulence, immune evasion, and molecular detections of SARS-CoV-2. Concomitantly, the importance of harnessing the immunobiology of SARS‐CoV‐2 and host interaction for therapeutic purpose; and use of an in slilico based biocomputational approaches to achieve this purpose *via* predicting novel therapeutic agents targeting PRR such as toll like receptor, design of universal vaccine and chimeric antibodies tailored to the emergent variant have been highlighted.

## Introduction

The disease COVID-19 came to prominence in December 2019 after many cases of pneumonia with unknown etiology appeared in Wuhan, China (Chen et al., 2020a; Huang et al., 2020a). Ultimately, the disease quickly spread to other provinces of China and the rest of the world (Chen et al., 2020a; Huang et al., 2020a). Due to its continuing spread, the WHO declared the outbreak as a public health emergency of international concern (Zarocostas, 2020). Subsequently, the virus was renamed as severe acute respiratory syndrome coronaviruse-2 (SARS-CoV-2) by the International Committee on Taxonomy of Viruses (Coronaviridae Study Group of the International Committee on Taxonomy of Viruses, 2020). To date, it has infected 604, 558, 779 million cases and killed 6,483,819 people worldwide (https://www.worldometers.info/coronavirus/). Coronaviruses (CoVs) are large, enveloped, single-stranded positive-sense RNA viruses belonging to the coronaviridae family (Chan et al., 2013; Ge et al., 2020). They have four genera namely alpha, beta, delta, and gamma CoVs (Hozhabri et al., 2020). Mammals are mainly infected by alpha and beta CoVs, and the gamma and delta CoVs primarily infect birds (Chen et al., 2020b; Ge et al., 2020; Wu et al., 2020). So far, seven CoVs had been identified causing disease in humans (Wu et al., 2020). The four human CoVs such as HCoV 229E, NL63, OC43, and HKU1 display endemic distribution globally resulting in mild upper respiratory tract infections and collectively are associated with 10%–30% cases of the common cold (Hozhabri et al., 2020; Wu et al., 2020). Conversely, SARS-CoV-2, MERS-CoV and SARS-CoV are the three CoVs that caused the most severe type of illness resulting in lower respiratory tract infections, acute respiratory distress syndrome (ARDS) and death in humans (Chen et al., 2020b; Paules et al., 2020). Accordingly, the case fatality rate of SARS-CoV is 10% whereas that of MERS-CoV is 37% (Huang et al., 2020b; Petersen et al., 2020). For SARS-CoV-2, several factors could affect the case fatality rate, making it a moving target. For instance, age variation, existence of comorbidity, capacity of health care systems, vaccinations, boosting, safety protocols are factors among others (Alimohamadi et al., 2021). Based on current estimates from a systematic review and meta-analysis, the overall estimated pooled case fatality rate of SARS-CoV-2 was 1.0% among the general population and is 19% in patients older than 50 years (Alimohamadi et al., 2021), making SARS-CoV-2 less deadly but more highly transmissible than its close relatives, SARS-CoV and MERS-CoV.

This review is centered on the SARS-CoV-2 genome structural organization, emerging genetic alterations that give rise to VOC, and features that distinguish them in terms of transmissibility, virulence, immune evasion, and the implications of this for diagnostic and therapeutic approaches.

### Origin and evolution of SARS-CoV-2, MERS-CoV, and SARS-CoV

Considering their origin and evolution, all CoVs initially existed in diverse species of bats as CoV-related viruses for example, SARS related CoV, MERS related COV and SARS related CoV2 (Ashour et al., 2020; Hozhabri et al., 2020; Lu et al., 2015). Subsequently, sequential mutations and recombination events allowed them to adapt to intermediate hosts for example, civets, camels, and pangolins, ultimately gaining access to human beings (Ashour et al., 2020; Hozhabri et al., 2020). For instance, MERS-CoV originated from bats and later adapted to dromedary camels as an intermediate host (Corman et al., 2014; Fung and Liu, 2019). Similarly, SARS-CoV adapted to civet and raccoon dogs as intermediate hosts. Thus, host jump (spillover) has contributed to the diversity and potential spillover of bat borne CoVs, where such zoonotic pathogens can cross species barriers to ultimately infect humans (Cui et al., 2019; Lu et al., 2015). In the case of SARS-CoV-2, given the challenge of identifying the origin and intermediate host as well as demand for several years of extensive work, utilizing the experience learned from the influenza virus is important (Gao et al., 2013). Moreover, collection of conclusive data in sufficient numbers in terms of genetic similarities and relevant geographical location provides better information (Wang et al., 2021a). In the end, a global search for sarbecoviruses by honing research on *Rhinolophus* bats, pangolins, and minks could be an effective strategy to identify intermediate hosts for SARS-CoV2 (Wang et al., 2021a; Zhao et al., 2020). When one considers that known coronaviruses are zoonotic viruses, these interspecies transmissions in CoVs can lead to evolution of another related novel CoVs (nCoVs) by jumping a natural host *via* a similar mechanism (spillover). Thus, although such undertakings require several years of continuous research, the accumulated data will provide a huge benefit for future origin-tracing endeavors (Gao et al., 2013; Wang et al., 2021a).

### Severe acute respiratory syndrome coronavirus 2 (SARS-CoV-2)

As noted, the SARS-CoV-2 is a novel zoonotic positive-sense RNA virus belonging to the genus beta coronaviruses in the Coronaviridae family (Chen et al., 2020c; Lu et al., 2020; Paraskevis et al., 2020). The SARS-CoV-2 has led to the outbreak of the disease COVID-19 and is the third CoVs causing pneumonia globally in the past 20 years (Ge et al., 2020). Clearly, the world has witnessed the devastating consequences of SARS-CoV-2 that has resulted in a global pandemic with unprecedented challenges and significant deaths worldwide. Central to the challenge posed by SARS-CoV-2 is its high transmission rate compared to its relatives SARS-CoV and MERS-CoV (Huang et al., 2020b; Petersen et al., 2020). However, SARS-CoV-2 is less deadly compared with the fatality rate of related species (Huang et al., 2020b; Petersen et al., 2020). Given the broad clinical spectrum and high transmission rate, and mutability of its genome leading to genetic alterations (VOC), eradicating, and managing the spread of SARS-CoV-2 pandemic will remain challenging in the foreseeable future (Petersen et al., 2020). As noted, information gained from genomic organization is indispensable as new variants caused by genetic alterations (mutations) are known to occur in viral infections (Grubaugh et al., 2020; Lauring and Hodcroft, 2021). Certainly, this is evident by the emergence of mutations leading to several new variants of SARS-CoV-2 (VOC) with the ability to spread worldwide. In addition to their complex genetic description reflecting genetic alteration, these emergent new variants are known by less complex WHO naming as Alpha, Beta, Gamma, and Delta which are previously circulating variants of concern (VOCs) (Figure 3), and Omicron variants, which is the current VOC (Figure 4). Thus, understanding the molecular signature of SARS-CoV-2 is important for laboratory identification, genetic surveillance, assessment of virulence, transmissibility, vaccine effectiveness and development of vaccines/drugs (combinatorial therapy).

### SARS-CoV-2 genome organization and expression

**FIGURE 1:**
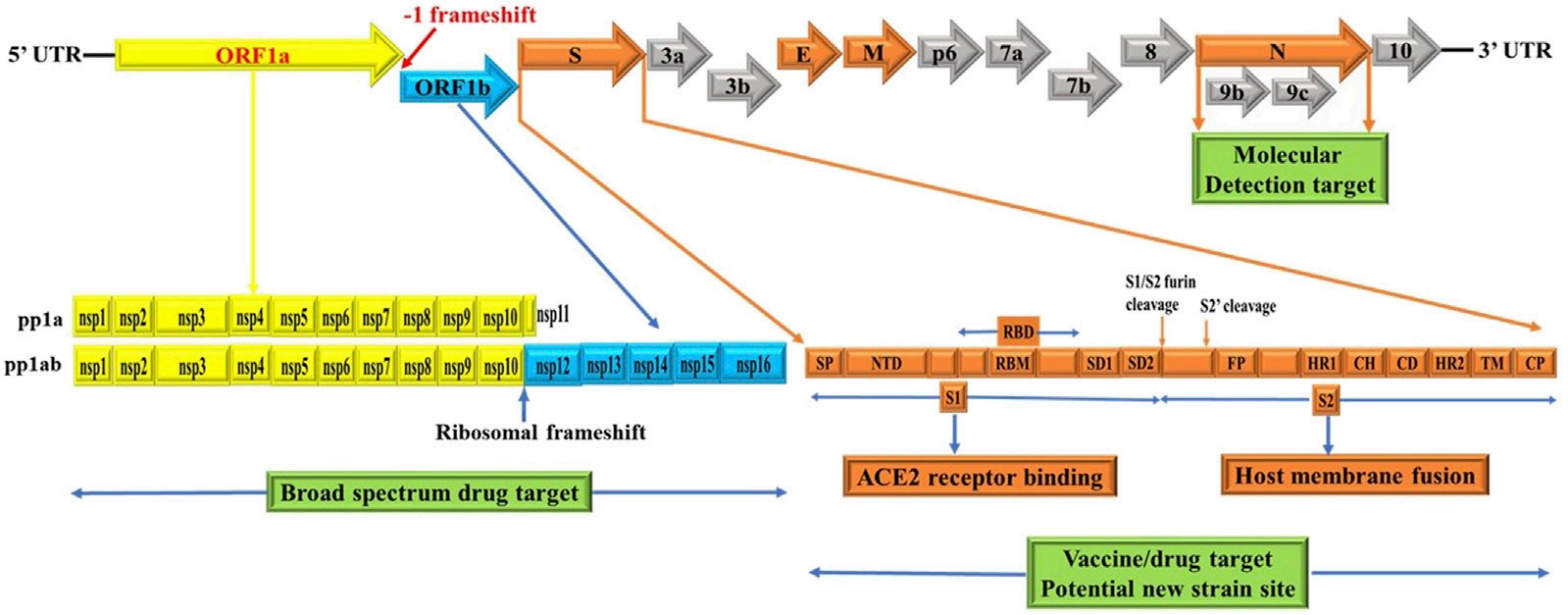
Schematics of genome structures of SARS-CoV-2. These schematics are based on information extracted from various studies (Baez-Santos et al., 2015; Berger and Schaffitzel, 2020; Cui et al., 2019; Chen et al., 2020b; Chen et al., 2020c; Chan et al., 2020; Ge et al., 2020; Hozhabri et al., 2020; Johnson et al., 2020; Michel et al., 2020; Peacock et al., 2021; Rabaan et al., 2020; Romano et al., 2020; Wu et al., 2020; Yoshimoto, 2020). Accordingly, the SARS-CoV-2 genome contains two untranslated regions (UTRs), one at 5′-cap structure and the second at the 3′-poly-A tail (Hozhabri et al., 2020). These two UTRs flank a single overlapping open reading frame (ORF) encoding a nonstructural polyprotein (Chan et al., 2020) followed by genes coding for four structural proteins, Spike (S), Envelope (E), Membrane (M), Nucleocapsid (N) and; sub group specific accessory genes coding for several accessory proteins, some of which are interspersed in the genes of structural proteins and even overlapping with structural genes (Chan et al., 2020; Ge et al., 2020; Hozhabri et al., 2020; Michel et al., 2020; Yoshimoto, 2020). SARS-CoV-2 spike glycoprotein domains (S domains) are shown in shades of orange scheme zoomed to magnify the detail. Accordingly, S1 comprises a signal sequence or peptide (SP), NTD, RBD, SD1 and SD2. S2 comprises a second furin cleavage site (S2′) upstream of the fusion peptide (FP), HR1, CH, CD, HR2, TM domain and cytoplasmic C-terminus peptide (CP) (Berger and Schaffitzel, 2020). Green box indicates targets for potential broad spectrum antiviral drugs, molecular detection targets, specific vaccines/drugs targets and genetic alteration hotspots (new variants sites). UTR: untranslated region, ORF: open reading frame, S: spike, E: envelope, M: membrane, N: nucleocapsid, NTD: N-terminal domain, RBD: receptor-binding domain, SD1: subdomain 1, SD2: subdomain 2, S2’: second protease (furin) cleavage site, FP: fusion peptide, HR1: heptad repeat 1, CH: central helix, CD: connector domain, HR2: heptad repeat 2, TM: transmembrane domain and CP: Cytoplasmic C-terminus peptide. Note: Furin cleavage sites are marked with brown arrow and green box show molecular detection target, vaccine/drug target and genetic alteration (new variant) site. The length of genes and proteins are not drawn to the scale. See text for additional information.

**FIGURE 2:**
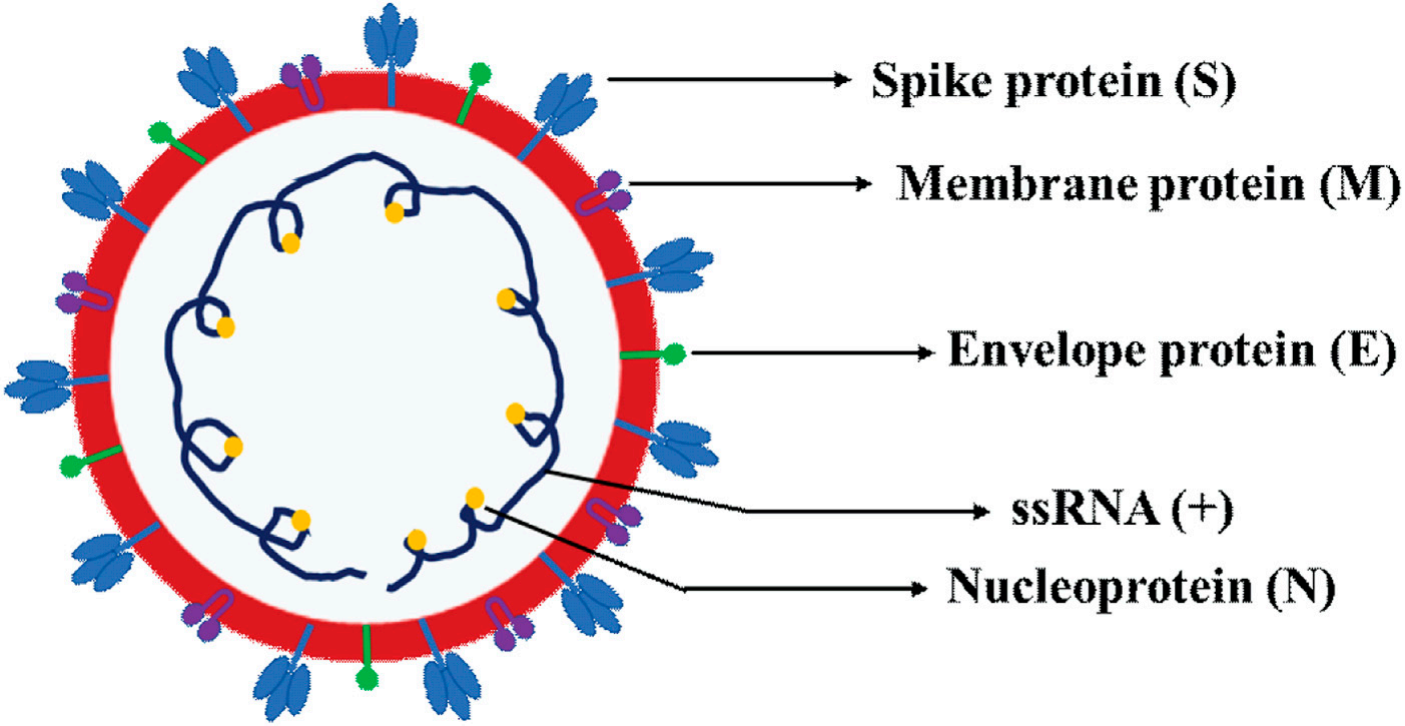
Schematic representation of SARS-CoV-2 virion structure. The schematics depicts the various four canonical structural proteins, namely the spike protein (S), envelope protein (E), membrane protein (M) and nucleoprotein (N) and single-stranded positive sense RNA (ssRNA+).

**FIGURE 3:**
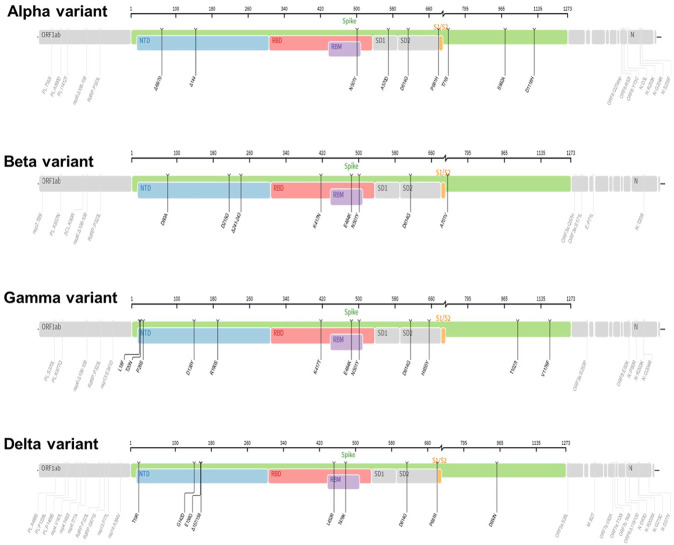
The mutation profiles of previously circulating different SARS-CoV-2 variants (VOCs). Accordingly, mutations profiles in the spike regions and other parts of SARS-CoV-2 genome are depicted. The mutation profiles are depicted from top to bottom in the order of Alpha, Beta, Gamma, and Delta VOCs. The pictures from covdb.stanford.edu are licensed under a Creative Commons Attribution-ShareAlike 4.0 International License https://creativecommons.org/licenses/by-sa/4.0/.

**FIGURE 4:**
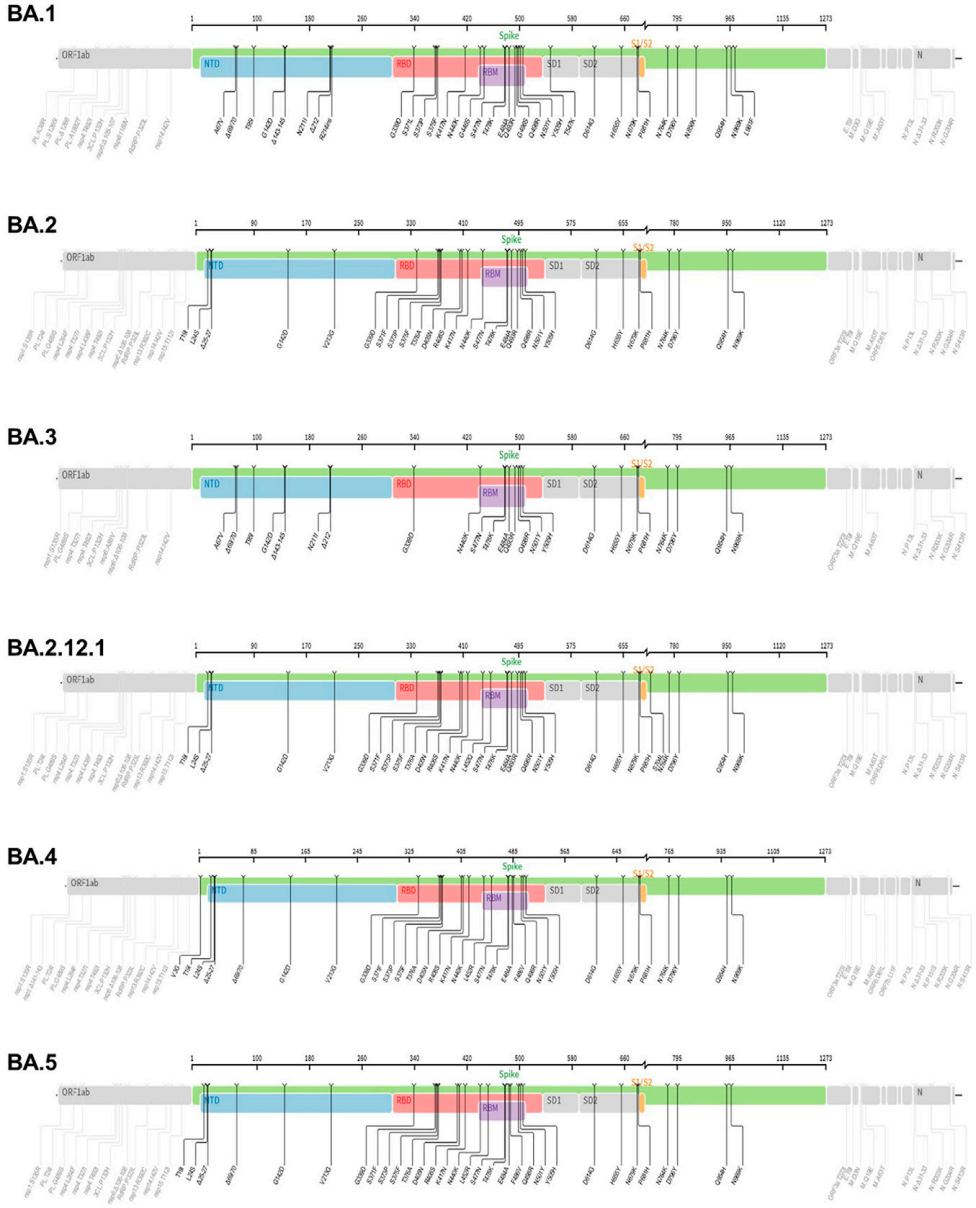
The mutation profiles of currently circulating Omicron variant (VOC) subvariants. Accordingly, mutations in the spike regions and other parts of SARS-CoV-2 genome are depicted. The mutation profiles are depicted from top to bottom in the order of BA.1, BA.2, BA.3, BA.2.12.1, BA.4 and BA.5 subvariants. he pictures from covdb.stanford.edu are licensed under a Creative Commons Attribution-ShareAlike 4.0 International License https://creativecommons.org/licenses/by-sa/4.0/.

The genome structure of SARS-CoV-2 comprises single-stranded positive sense RNA (ssRNA+) genome that is around 29.8 kb, providing a trove of information on its genome sequences (Hussain et al., 2020) and Figure 1. This genetic information is central to understanding SARS-CoV-2 evolution, pathogenesis, and the emergence of new variants which occur *via* mutation mediated genetic alterations (Figures 3, 4). The genetic information is particularly important for identification and monitoring of resistant variants and the development of new vaccines tailored to variant epitope, therapeutic monoclonal antibodies (designing chimeric antibodies), and antiviral drugs (combinatorial therapy). In addition, the information gained from the genomic structure is crucial for the molecular diagnostic-based detections. For example, current real time RT-PCR nucleic acid detection is based on the information extracted from specific regions targeting the conserved regions of S, E, N, nsp12, nsp14 and ORF1ab in the genome of novel SARS-CoV-2, which are key for the design of specific primers employed for accurate and specific detection. As shown in Figure 1, the SARS-CoV-2 genome is schematically represented in the pattern of 5′-UTR-ORF1a (yellow), ORF1b (blue), structural proteins (S, E, M, N, orange), accessory genes scattered among structural genes (gray) and 3′-UTR. Typically, the genome of SARS-CoV-2 entails two untranslated regions (UTRs) as 5′-cap structure and 3′-poly-A tail, which are located at two extreme ends (Hozhabri et al., 2020). These two UTRs flank a single, large overlapping open reading frame (ORF) encoding a nonstructural polyprotein (Chan et al., 2020) followed by genes coding for four structural proteins including Spike (S), Envelope (E), Membrane (M), Nucleocapsid (N) (Figure 2), and accessory genes coding for accessory proteins, some of which are interspersed in the genes of structural proteins and even overlapping with structural genes (Chan et al., 2020; Ge et al., 2020; Hozhabri et al., 2020; Michel et al., 2020; Yoshimoto, 2020).

### SARS-CoV-2 ORF1a and ORF1b genes

The ORF1a (yellow) and ORF1b (blue) are two large genes that are translated into polypeptides 1a (PP1a) (yellow) which is short; and PP1ab (yellow/blue) which is long nonstructural proteins (NSP) (Chan et al., 2020; Ge et al., 2020; Hozhabri et al., 2020; Michel et al., 2020; Yoshimoto, 2020) (Figure 1). Of note, the first ORFs of SARS-CoV-2 (ORF1a and ORF1b) comprise large portion (2/3) of the genome length and encode for 16 nonstructural polyproteins (NSPs 1–16), which are directly translated from genomic RNA (Cui et al., 2019). In this regard, it is a -1 ribosomal frameshift located between ORF1a and ORF1b that is responsible for the production of these two large replicase polypeptides (PP1a and PP1ab), key proteins that are important for viral replication (Hozhabri et al., 2020; Michel et al., 2020). Functionally, two virally encoded cysteine proteases, papain-like protease (PLpro) and 3-chymotrypsin-like protease (3CLpro) are key players for further processing of PPs into 16 NSPs (NSPs 1–16) (Baez-Santos et al., 2015; Chen et al., 2020b; Romano et al., 2020). For instance, the NSP14 of CoVs functions as proofreading machinery for prevention of lethal mutagenesis using its N-terminal exoribonuclease (ExoN) domain, and the C-terminal domain as a (guanine-N7) methyl transferase (N7-MTase) for mRNA capping (Ma et al., 2015; Ogando et al., 2020). Given the importance of these proteases and NSP14 (ExoN) for viral replication and transcription which ensures viral propagation, they can be targeted to develop potential broad-spectrum antiviral drugs (pan antiviral drugs) (Ma et al., 2015; Ogando et al., 2020) (Figure 1, green box) and for detection purposes (reviewed in molecular detection section). For example, Remdesivir inhibits RNA-dependent RNA polymerase (RdRp) of CoVs including SARS-CoV-2 by acting as a nucleoside analog; and is FDA-approved for the treatment of COVID-19 patients (Kokic et al., 2021). Further, all CoVs, including SARS-CoV-2, encode two proteases required for PP1A and PP1AB polyproteins processing (Figure 1 yellow and blue) (Reina and Iglesias, 2022). In this context, the key protease chymotrypsin-like (3CL) catalyze the processing of NSP11/16 proteins and is targeted for the development of SARS-CoV-2 antiviral drugs due to the high sequence and structure conservation among all CoVs (Reina and Iglesias, 2022). One such example of an antiviral drug targeting a key viral replication machinery (protease) is Paxlovid (nirmatrelvir and ritonavir) from Pfizer (Reina and Iglesias, 2022) which has received emergency use authorization (EUA) by FDA. Similarly, Molnupiravir from Merck increases the frequency of viral RNA mutations by targeting viral RNA-dependent RNA polymerase (RdRp) and impairs SARS-CoV-2 replication (Kabinger et al., 2021) and has received EUA by FDA.

### Significance of -1 ribosomal frameshift

As in dictated in Figure 1, the synthesis of PP1ab involves a -1 ribosomal frameshift that occurs in the overlapping region between ORFs1a and ORFs1b. The -1 ribosomal frameshift occurs during the translational elongation step which is required for the expression of protein from CoVs gene1, the largest gene accounting for 2/3 of the CoVs genome (Bekaert and Rousset, 2005; Nakagawa et al., 2016). Thus, PP1ab is encoded by fusion from the two ORFs (ORF1a and ORF1b (Chan et al., 2020; Ge et al., 2020; Hozhabri et al., 2020; Michel et al., 2020; Yoshimoto, 2020). Functionally, most of the mature NSPs produced from these two large PPs are important for synthesizing CoVs RNA (Bekaert and Rousset, 2005; Nakagawa et al., 2016). Thus, -1 ribosomal frameshifting dependent translation of PP1ab is an important step for replication of COVs (Bekaert and Rousset, 2005; Nakagawa et al., 2016). Consistent with this, reduced frameshifting efficiency that affects ratio of PP1a/PP1ab is reported to impact virus infectivity and replication (Ishimaru et al., 2013). As CoVs have evolved to produce optimal levels of -1 ribosomal frameshift for efficient replication (Bekaert and Rousset, 2005; Ishimaru et al., 2013; Nakagawa et al., 2016), it is an evolutionally conserved features of CoVs including SARS-CoV-2, SARS-CoV and MERS-CoV.

### SARS-CoV-2 canonical structural genes

Of particular note, the subgenomic RNAs is translated to give rise to all structural and accessory proteins during the transcription/replication of the CoVs genome (Fung and Liu, 2019; Kim et al., 2020). This contrasts with SARS-CoV-2 ORF1a and ORF1b proteins which are directly translated from the RNA genome (Cui et al., 2019). Accordingly, the 3′-end of SARS-CoV-2 genomes contain four canonical structural proteins (orange), namely the S, E, M and N proteins, as common entity to all CoVs (Chen et al., 2020b) and (Figure 2). Of note, the M protein is higher in quantity than any other protein in the virus particle (Masters, 2006; Nal et al., 2005). Functionally, the M protein shapes the virions, promotes membrane curvature, and binds to the nucleocapsid using its three transmembrane domains (Masters, 2006; Nal et al., 2005). Conversely, the N protein binds to NSP3 protein using its two domains which help tether the genome to replication/transcription complex and allow viral RNA packaging into viral particle during viral assembly (Masters, 2006; Hurst et al., 2009). For molecular diagnostics, it is imperative to have accurate genetic information regarding the target gene in the SARS-CoV-2 genome. Thus, the N protein has significance pertaining to diagnosis. For example, the N gene encoding for N protein has been targeted and is in use to provide much needed information for the design of SARS-CoV-2 specific primers (https://www.raybiotech.com/coronavirus-nucleic-acid-detection-kit/\) and (Figure 1, green box)**.** Further, the N protein is abundantly expressed during infections and has high immunogenic activity. While the E protein is key player for the assembly of the virus and release of the virion from host cells, the S protein has cardinal role in the attachment to host cell receptors, viral entry and determines host tropism (Du et al., 2009; Siu et al., 2008) and Figure 6. Significantly, the S protein is the target for vaccine/drug development, and virtually all emergent new variants have their genetic alterations (mutations) in the spike protein, making it an indispensable part of the SARS-CoV-2 genome for clinical, molecular detection, therapeutic intervention, epidemiological and research purposes.

### SARS-CoV-2 spike genes

Apparently, the S glycoprotein is of particular interest because it is a target for vaccine/drug design, development, and production as well as molecular detection. Interestingly, SARS-CoV-2 has a high transmission rate and is prone to genetic alteration at the S gene site coding for S glycoprotein, despite the similarity between SARS-CoV and MERS-CoV (Rabaan et al., 2020; Davies et al., 2021). In this context, the SARS-CoV-2 utilizes its S proteins to bind to angiotensin-converting enzyme 2 (ACE2) and infect human cells (Chen et al., 2020d; Ge et al., 2020; Huang et al., 2020b) and Figure 6. Of note, the S protein of SARS-CoV-2 contains two regions, S1 subunit and S2 subunit, having a size of 180–200 kd (Huang et al., 2020b; Ge et al., 2020) and (Figure 1, orange zoomed to show detail). As shown in Figure 6 depicting host virus interaction, there are two furin (protease) cleavage sites for S protein, one located at the S1/S2 interface and the other at the S2’ position of the S protein, and these are key to viral replication and pathogenesis (Johnson et al., 2020; Peacock et al., 2021). The cleavage of S glycoprotein into S1 and S2 subunits enables fusion of the virus with the host cell membranes (Chen et al., 2020d; Ge et al., 2020; Huang et al., 2020b) and Figure 6. Given that furin cleavage sites facilitate priming that might increase the spread of SARS-CoVs-2, furin inhibitors can be targeted as potential drug therapies for SARS-CoV-2 (Rabaan et al., 2020). Of particular interest, Deletion of H69/V70 at the spike gene enhances infectivity of SARS-CoV-2 *in vitro* and is associated with immune escape in immunocompromised patients (Davies et al., 2021). Further, the current omicron variant has three mutations at the furin cleavage site (Chen et al., 2021; Chavda and Apostolopoulos, 2022) a site known to increase SARS-CoV-2 infectivity (Chen et al., 2021; Chavda and Apostolopoulos, 2022) and Figure 4. Consistent with these mutations, omicron variant has an increased risk of reinfection compared to predecessor variants (Chen et al., 2021; Chavda and Apostolopoulos, 2022). Generally, the S1 domain is associated with receptor binding and the S2 domain with cell membrane fusion (Chen et al., 2020d; Ge et al., 2020; Huang et al., 2020b) and (Figure 6). Consistent with this, S1 contains N-terminal domain (NTD) and a receptor-binding domain (RBD) which harbors core domain and external subdomain (ESD) (Huang et al., 2020b). S2 contains three functional domains, fusion peptide (FP), and heptad repeat (HR) one and HR2 (Chen et al., 2020d; Ge et al., 2020; Huang et al., 2020b) and (Figure 6). Thus, whether SARS-CoV-2 can combine with host cells or not is determined by the affinity between viral RBD and the ACE2 receptor of human cells (Huang et al., 2020b; Ge et al., 2020). Once RBD binds to the receptor in the initial stage, then S2 changes conformation, facilitating membrane fusion by its three functional domains (FP, HR1 and HR2) (Chen et al., 2020d; Ge et al., 2020; Huang et al., 2020b). Interestingly, SARS-CoV-2 and SARS-CoV have about 75% amino acid identity in their S protein (Chen et al., 2020d; Ge et al., 2020; Huang et al., 2020b). For instance, the S1 functional domain (RBD) of SARS-CoV-2 has 72%–74.9% identity of amino acid sequences in both viruses, making it a close relative to SARS-CoV (Chen et al., 2020d; Ge et al., 2020; Huang et al., 2020b). As for the functional domains of S2, there is no appreciable difference between SARS-CoV-2 and SARS-CoV except for some non-critical amino acid residues in HR1 region (Ge et al., 2020; Wan et al., 2020). These similarities prompt caution for designing unique primers that do not overlap with these similar regions in the spike gene.

### SARS-CoV-2 ACE2 receptor

As noted, SARS-CoV-2 infects human cells such as the alveolar endothelium in the lung by binding to ACE2 receptors, membrane receptor (Ge et al., 2020; Yan et al., 2020; Yang et al., 2020) and **(** Figure 6 **)**. Mechanistically, this binding results in endocytosis of the viral complex with consequent local activation of the renin angiotensin aldosterone system (RAAS), resulting in acute lung injury that may progress to acute respiratory distress syndrome (ARDS) in humans (Ge et al., 2020; Yan et al., 2020; Yang et al., 2020). Mounting data show that several critical residues in SARS-CoV-2 RBD have good interactions with human ACE2 receptors (Chen et al., 2020d; Ge et al., 2020; Huang et al., 2020b; Yan et al., 2020). Most residues of the RBD that interact with ACE2 are fully conserved (Chen et al., 2020d; Ge et al., 2020; Huang et al., 2020b). Other evidence supporting ACE2 as a receptor of cells is that the HR1 and HR2 domain of SARS-CoV-2 can fuse with each other to form a 6-helical bundle (6-HB) akin to SARS-CoV’s fusion mechanism (Ge et al., 2020; Xia et al., 2020). Evidence from studies that utilized structural analysis documented that SARS-CoV-2 strongly interacted with ACE2 compared with SARS-CoV (Chen et al., 2020d; Ge et al., 2020). By elucidating the cryo-EM structure of SARS-CoV-2 S protein, it was documented that SARS-CoV-2 bound to ACE2 with 10–20-fold higher affinity than SARS-CoV (Wrapp et al., 2020). Interestingly, the existence of six mutations in the receptor-binding motif (RBM) conferred SARS-CoV-2 S glycoprotein a higher affinity for ACE2 than SARS-CoV (Berger and Schaffitzel, 2020), suggesting mutation induced gain of function (accounting for higher transmission rate of SARS-CoV-2) (Berger and Schaffitzel, 2020; Walls et al., 2020; Wrapp et al., 2020). Mechanistic evidence informs that ACE2 binding triggers conformational changes promoting proteases to further cleave S2, followed by shedding of S1 and activation of S2 refolding into a post-fusion state (Cai et al., 2020). This is promoted by the acquisition of a furin cleavage site between S1 and S2, a feature important for pathogenicity of SARS-CoV-2 (Wrobel et al., 2020) and Figure 6.

### SARS-CoV-2 accessory genes

All accessory proteins are translated from subgenomic RNAs (Fung and Liu, 2019; Kim et al., 2020). Functionally, SARS-CoV-2 accessary proteins plays a role for viral release, stability, pathogenicity, and virulence but are not necessary for virus replication (Baruah et al., 2020). Notably, the genes encoding accessory proteins are distinct in different CoVs in terms of number, genomic organization, sequence, and functions (Hozhabri et al., 2020; Song et al., 2019; Wu et al., 2020; Wu and McGoogan, 2020). Accordingly, the 3′-end of the genome of SARS-CoV-2 harbor nine accessory proteins [3a, 3b, p6, 7a, 7b, 8, 9b and 9c (ORF14) early study and ORF10] (Michel et al., 2020; Yoshimoto, 2020) and along with structural proteins (S, E, M and N). In addition, the accessory proteins in CoVs also vary in location and size in different viral subgroups (Ge et al., 2020; Hozhabri et al., 2020; Michel et al., 2020). For example, SARS-CoV-2 and SARS-CoV are significantly different in terms of gene sequence of two accessory proteins (ORF3b and ORF8) (Chen et al., 2020c; Ge et al., 2020; Michel et al., 2020; Romano et al., 2020). In addition, the ORF10 accessory gene is proposed as unique to SARS-CoV-2 and located downstream of N gene and codes for a 38 amino acid long peptide (Michel et al., 2020; Yoshimoto, 2020). This information is critical for designing unique primers for detection purposes.

### SARS-CoV-2 genetic variants

The emergence of new genetic variants of SARS-CoV-2 has spurred intense interest among scientific, clinical, and public health experts while creating anxiety for the population at large. Thus, to understand and identify the variants of concern, information inferred from genomic organization is key. It also helps to understand virus evolution and the genomic epidemiology of SARS-CoV-2 (Galloway et al., 2021; Lauring and Hodcroft, 2021). In this context, it is worth noting that viral mutations (genetic alterations) are not uncommon and occur as a natural consequence of viral replication (Grubaugh et al., 2020; Lauring and Hodcroft, 2021; Mohammadi et al., 2021). Typically, when compared with DNA viruses, RNA viruses tend to have higher rates of mutation (Akkiz, 2021; Lauring and Hodcroft, 2021; Mohammadi et al., 2021). Conversely, owing to their ability to encode an enzyme that corrects some of the errors made during replication, CoVs generate fewer mutations than most RNA viruses (Akkiz, 2021; Lauring and Hodcroft, 2021; Mohammadi et al., 2021). Generally, once genetic alterations occur, natural selection dictates the fate of a newly arising mutation (Akkiz, 2021; Lauring and Hodcroft, 2021; Singh and Yi, 2021). For example, while those mutations that reduce viral fitness will diminish the population of incompetent viruses, those mutations that impart a competitive advantage related to viral replication, transmission, or escape from host immunity, will increase within a population. (Lauring and Hodcroft, 2021). Consequently, virus evolution and spread within hosts, in communities, and across countries are shaped at least in part by the natural selection (26,67,68, (Singh and Yi, 2021). Thus, genomic organization of SARS-CoV-2 helps to provide useful genomic information related to evolution of a new SARS-CoV-2 variant and which mutations are being enriched to allow increased circulation in the population (Akkiz, 2021; Lauring and Hodcroft, 2021; Mohammadi et al., 2021). Of particular interest is the identification that these variants have a high number of mutations in the S protein within the amino terminal domain (NTD) and Receptor-binding domain (RBD). These mutations have a direct influence on viral infection rate by enhancing the affinity of RBD for the ACE2 receptor (Akkiz, 2021; Grubaugh et al., 2020; Gomez et al., 2021; Lauring and Hodcroft, 2021; Singh and Yi, 2021) and (Figures 3, 4). Comprehending the adaptive benefit of these mutations on transmissibility, antigenicity, or virulence is of primary importance in any effort geared towards intervention and eventual quelling of community spread (Akkiz, 2021; Grubaugh et al., 2020; Gomez et al., 2021; Lauring and Hodcroft, 2021; Singh and Yi, 2021). In this context, genomic surveillance and laboratory experiments are important players during the emergence of new stains as the former provide information regarding which mutations are emerging, and later can help determine how these mutations change the virus from its predecessor (original virus) in terms of transmissibility, virulence, host evasion, response to interventional therapy and detection (Grubaugh et al., 2020; Lauring and Hodcroft, 2021). In the end, these tools help us identify and comprehend which variants are of greatest concern. Accordingly, several notable variants of concern (VOC) have been identified and are being monitored for implementation of public health strategies that mitigate transmission risks. Next, we will review previously circulating VOC such as Alpha, beta, gamma, and delta (Figure 3), and recently circulating VOC notably omicron and its subvariants (Figure 4).

### Previously circulating SARS-CoV-2 variants of concern (VOCs)

#### Lineage B.1.1.7 also called 501Y.V1 (alpha variant)

The lineage B.1.1.7 also known as 501Y.V1 (Alpha Variant, WHO naming) was a variant of concern (VOC), identified initially in United Kingdom in September 2020. It harbors several mutations in the spike glycoprotein and other sites such as OR1F1ab, Orf8 and N (Grubaugh et al., 2020; Gomez et al., 2021; Lauring and Hodcroft, 2021; Mohammad et al., 2021) and Figure 3. For example, the N501Y mutation occurs in the Receptor-binding domain (RBD) whereas the P681H occurs near the S1/S2 furin cleavage site, a site with high variability in CoVs (Grubaugh et al., 2020; Gomez et al., 2021; Lauring and Hodcroft, 2021) and a potential therapeutic drug target (Rabaan et al., 2020). Data show that acquisition of a P681H mutation at the furin site enhance cleavage of Spike protein (Davies et al., 2021; Shrestha et al., 2022). Further, report from structural modelling of SARS-CoV-2 documented alpha variant having an enhanced furin binding and infectivity (Mohammad et al., 2021). Given the 501Y spike variants are predicted to have a higher affinity for human ACE2 receptors, conceivably, these mutations could potentially influence ACE2 binding and viral replication (Grubaugh et al., 2020; Gomez et al., 2021; Lauring and Hodcroft, 2021; Mohammad et al., 2021), conferring competitive fitness for community spread. Consequently, the expansion of lineage B.1.1.7 was linked to wide spread of SARS-CoV-2 cases, achieving dominance by outcompeting an existing population of circulating variants (Davies et al., 2021; Grubaugh et al., 2020; Gomez et al., 2021; Lauring and Hodcroft, 2021). For instance, population genetic models suggested that lineage B.1.1.7 was spreading 56% more quickly than other lineages (Davies et al., 2021; Lauring and Hodcroft, 2021). Despite the quick spread, evidence showed that vaccines remained effective againist this variant lineage (Lauring and Hodcroft, 2021; Wu et al., 2021; Xie et al., 2021).

#### Linage B.1.351 also called 501Y.V2 (Beta Variant)

The linage B.1.351 also known as 501Y.V2 (Beta Variant, WHO naming) was another variant of concern (VOC) that was detected initially in August 2020 in South Africa. It harbors multiple mutations in the spike protein notably, changes in the RBD site, K417 N/T, E484K, and N501Y as well as other part of the genome, OR1F1ab, Orf3a, E and N (Galloway et al., 2021; Gomez et al., 2021; Hoffmann et al., 2021) and Figure 3. Based on evidence from reduced neutralization by convalescent and post-vaccination sera, studies indicate that one of the spike protein mutations, E484K may affect neutralization capacity of some polyclonal and monoclonal antibodies (Weisblum et al., 2020; Wang et al., 2021b). Further, the linage B.1.351 evade infection induced antibody responses, certain therapeutic antibodies, and vaccination (Hoffmann et al., 2021). Specifically, acquisition of E484k mutation conferred resistance to NTD raised most mAbs and RBD motif raised multiple individual mAbs (Wang et al., 2021b). In addition, B.1.351 is 9.4-fold more resistant to neutralization by convalescent plasma and 10.3–12.4 fold to sera from vaccinated individual (vaccinee sera) (Wang et al., 2021b).

#### Lineage P.1 also called 501Y.V3 (gamma variant)

The lineage P.1 also known as 501Y.V3 (Gamma variant, WHO naming) was identified initially in July 2020 in Brazilian travelers to Japan. It acquired mutations in the Receptor-binding domain of spike proteins notably, K417 N/T, E484K, and N501Y as well as another site of the genome including, OR1F1ab, Orf3a, Orf8 and N (Hoffmann et al., 2021) and Figure 3. This variant was later identified as the dominant variants in Brazil and was subsequently detected in the USA and several other countries (Galloway et al., 2021; Gomez et al., 2021). The emergence of the gamma variant raised concerns owing to increased transmission, propensity for re-infection (Resende et al., 2021) and reduced neutralization by convalescent and post-vaccination sera (Wang et al., 2021c). Report showed that linage P.1 evaded antibody responses to infection, certain therapeutic antibodies, and vaccination (Hoffmann et al., 2021; Wang et al., 2021b).

#### Lineage B.1.617.2 (delta variant)

The lineage B.1.617.2 (Delta variant, WHO naming) was another variant of concern (VOC) that was initially identified in December 2020 in India, with subsequent detection in the United States, and worldwide distribution (Lopez Bernal et al., 2021; Singh et al., 2021). This variant has acquired distinct mutations in the spike protein including P681R, L452R and D950N as well as mutations on another site of the genome, OR1F1ab, Orf3, M, Orf7a, Orf8 and N (Mlcochova et al., 2021; Zhan et al., 2022), Figure 3. Interestingly, it lacks E484K, and N501Y mutation which are shared by other viral strains (Zhan et al., 2022). Notably, Delta variant has a higher transmission rate and immune evasion capacity (Lopez Bernal et al., 2021; Singh et al., 2021). As noted, the furin cleavage site is important for determining fusion of the virus with the host cells (Figure 6). Report show that acquisition of a P681R mutation at the furin site enhances cleavage of the Spike protein (Shrestha et al., 2022). Data from a study looking into effectiveness of COVID-19 vaccines against the B.1.617.2 (Delta variant) showed lower efficacy of BNT162b2 or ChAdOx1 nCoV-19 vaccines among persons with the Delta variant than among those with the Alpha variant after receiving single vaccine doses (Lopez Bernal et al., 2021). Further, an *in vitro* study revealed that the delta variant is 6-fold less sensitive to serum neutralizing antibodies obtained from recovered individuals, and 8-fold less sensitive to antibodies elicited from vaccine, in comparison to wild type (Mlcochova et al., 2021). Next, we will review the VOC currently in circulation.

#### Lineage B.1.1.529 (omicron variant)

The lineage B.1.1.529 (Omicron variant, WHO naming) is the recent variant of concern (VOC) that was reported initially on 24 November 2021from South Africa (Chavda and Apostolopoulos, 2022). Evaluation of the evolution of SARS-CoV2 using a sequence-based approach revealed that B.1.1.529 variants have acquired many mutations, implicating omicron variant as the most highly mutated strain among all SARS-CoV-2 variants (Nie et al., 2022; Teng et al., 2021; Tuekprakhon et al., 2022). As depicted in Figure 4, the Omicron variant has significantly more missense mutations accumulated in spike and other genome regions compared to the predecessor VOCs, Alpha, Beta, Gamma, and Delta variants (Figure 4). For example, more than 60 mutations have been identified on several genomic regions of the virus namely, Spike protein, ORF1ab, Envelope, Membrane, Nucleocapsid proteins (Nie et al., 2022). Notably, the spike protein which is the antigenic target for antibody production by infected host and vaccines have been targeted by 26–35 mutations which are unique (nonsynonymous) from the original SARS-CoV-2 variants (Ren et al., 2022). Of note, omicron has high binding affinity to ACE2 receptor due to acquisition of substitutions mutations such as Q493R, N501Y, S371L, S373P, S375F, Q498R, and T478K at the RBD site (Shrestha et al., 2021; Araf et al., 2022) and Figure 4. Additionally, the variant has three mutations at the furin cleavage site, namely N679K, H655Y, and P681H (Chen et al., 2021; Chavda and Apostolopoulos, 2022), a site known to increase SARS-CoV-2 infectivity (Chen et al., 2021; Chavda and Apostolopoulos, 2022). Consistent with these mutations, the Omicron variant has an increased risk of reinfection compared to predecessor variants (Chen et al., 2021; Chavda and Apostolopoulos, 2022). For instance, Omicron spread faster than the Delta variant albeit causing less severe disease (Ren et al., 2022).

Conceivably, the high rate of spread, ability to evade double vaccination, and the host immune system can increase the burden on the health care systems, even though Omicron infections are less fatal than infections with the Delta variant (Cao et al., 2022a; Ren et al., 2022). To counter the burden posed by Omicron, vaccination with a third dose of mRNA vaccine can provide protection against severe disease and hospitalization by supercharging the neutralizing antibody levels and strengthening host immunity (Ren et al., 2022). Further, extending vaccine boosting efforts across different age groups can mitigate the strain on the health care system as evidenced by the BNT162b2 vaccine conferring protection against Omicron variant in children and adolescents (Price et al., 2022).

### The Omicron subvariants

The Omicron (B.1.1.529) variant has several subvariants, namely BA.1 (B.1.1.529.1), BA.2 (B.1.1.529.2), BA.3 (B.1.1.529.3), BA.2.12.1, BA.4 and BA.5 (Desingu and Nagarajan, 2022; Majumdar and Sarkar, 2022). They share many of the mutations while also being significantly different (Desingu and Nagarajan, 2022; Majumdar and Sarkar, 2022). Interestingly, these subvariants were detected in South Africa indicating fast evolutionary divergence of the subvariants (Rahimi and Talebi Bezmin Abadi, 2022). Importantly, the existence of many mutations on several regions of the genome (Figure 4) raises questions as to why such genomic instability is occurring in a virus that has an error correcting (proof reading) exonuclease (the nsp14 protein) (Chen et al., 2021; Gribble et al., 2021). The nsp14 protein mediate viral recombination and is highly conserved among CoVs (Gribble et al., 2021). Despite its role contributing to a low mutation rate and stability of the viral genome, more than six million viral genomes have been documented, suggesting the instability of SARS-CoV-2 genome (Eskier et al., 2020; Thakur et al., 2022). Consistent with this and as shown in Figure 4, the diverse Omicron subvariants (BA.1, BA.2, BA.3, BA.2.12.1, B.4 and BA.5) harbor nsp14 mutations (Figure 4). Further, data reveal mutations in SARS-CoV-2 nsp14 have the strongest association with increased mutation load across the genome compared to nsp7, nsp8 and nsp12 which form the core polymerase complex (Eskier et al., 2020). As discussed above, these subvariants are distinct. For instance, evidence reveals that BA.2 is more transmissible and may cause more severe disease than BA.1 ((Rahimi and Talebi Bezmin Abadi, 2022), (Chen and Wei, 2022)). Further BA.2 is not affected by the therapeutic monoclonal antibodies used to treat people infected with COVID, making BA.2 more resistant to casirivimab, imdevimab and sotrovimab monoclonal antibodies than the original Omicron variant (B.1.1.529) (Iketani et al., 2022) suggesting the fitness of BA.2 subvariant.

The BA.2.12.1 subvariant is diverged from BA.2 by acquiring additional spike mutations S704L and L452Q on top of BA.2 background and causing breakthrough infections in fully vaccinated and boosted individuals (Beheshti Namdar and Keikha, 2022). For example, prior infections with BA.1 appear to confer minimal cross immunity to BA.212.1. As a result, an individual with BA.1 infection can also be infected with BA.2.12.1 (Cao et al., 2022b; Del Rio and Malani, 2022). Early studies document that BA.2.12.1 is about 25% more transmissible than BA.2 but does not appear to cause severe disease (Beheshti Namdar and Keikha, 2022). Consequently, approximately 58% of SARS-CoV-2 isolates sequenced belong to BA.2.12.1 as of May 2022 (Del Rio and Malani, 2022), suggesting fitness of this subvariant.

**FIGURE 5:**
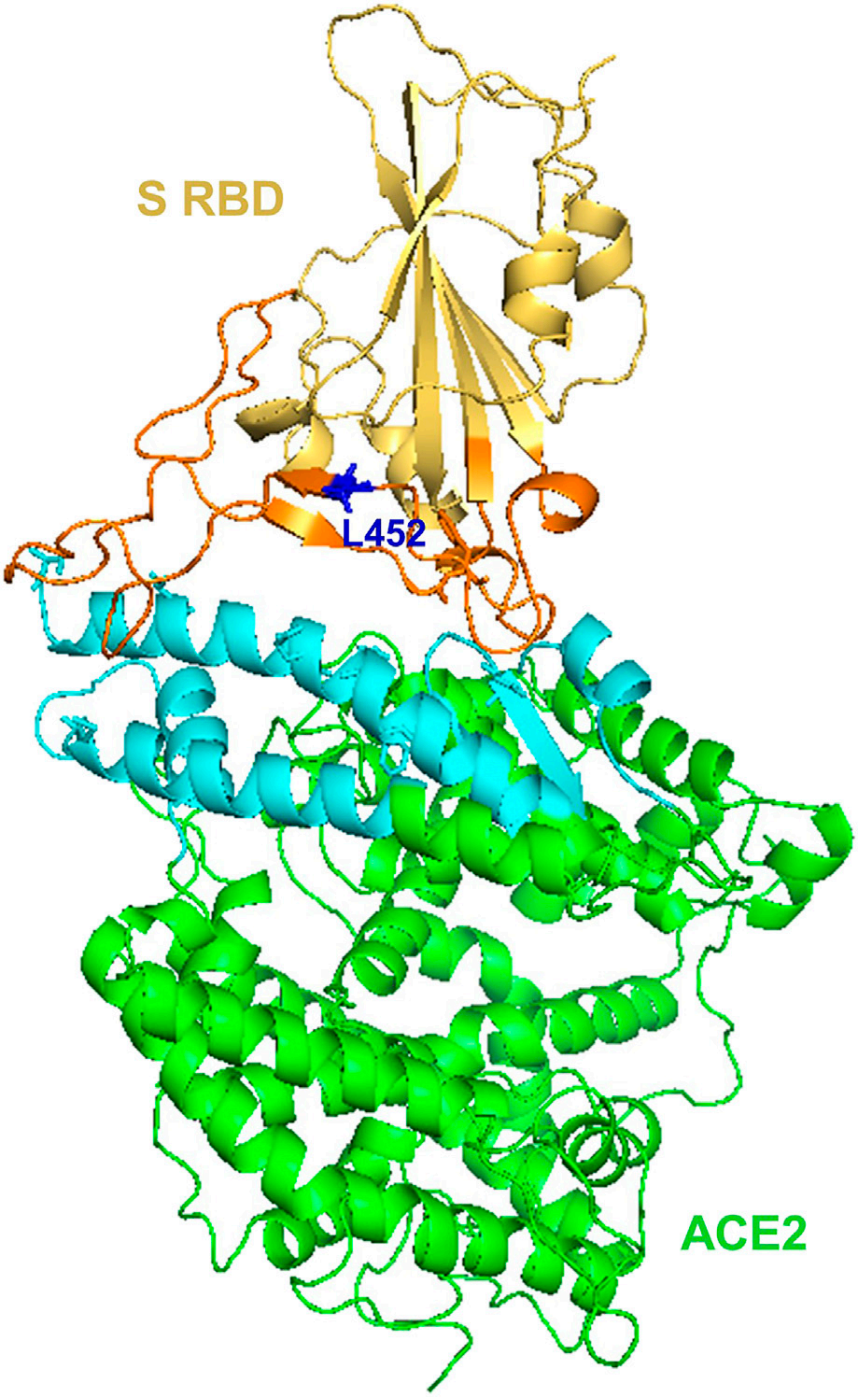
Structural representation of SARS-Cov-2 Spike and human ACE2 proteins. SARS-CoV-2 spike receptor-binding domain (RBD, yellow) and its interaction regions (orange), Human receptor ACE2 (green) and its interaction regions (cyan) were displayed as cartoons. SARS-CoV-2 spike L452 residues (blue) was shown as sticks. The figure was generated using PyMOL (http://www.pymol.org/) based on PDB ID: 6LZG.

The BA.4 and BA.5 subvariants also resemble more of BA.2 than BA.1 and emerged in south Africa and Europe displaying competitive advantage in terms of viral replication, transmissibility, and immune evasion (Del Rio and Malani, 2022; Tuekprakhon et al., 2022). Particularly, the acquisition of L452R and F486V substitution mutation confer BA.4 and BA.5 the ability to have increased ACE2-binding affinity, stronger neutralization evasion, as well as higher transmissibility than BA.2 (Del Rio and Malani, 2022; Tuekprakhon et al., 2022). Given the potential of the 69-70del, L452R, and F486V substitution mutations to alter the binding affinity between SARS-CoV-2 spike and human ACE2 (Teng et al., 2021; Tuekprakhon et al., 2022), we have performed an *in silico* analysis to investigate the effects of viral variations on spike-ACE2 interaction. We showed that some mutations may enhance the binding affinity (Teng et al., 2021). For example, L452R is located in the interface of spike-ACE2 complex (Figure 5). We observed that L452R can increase the binding affinity (ΔΔΔG = −0.395 kcal/mol) between SARS-CoV-2 spike and human ACE2 (Teng et al., 2021). Consistent with our finding, L452R mutation was identified in Omicron BA.4/5 variant and found to play a critical role in the immune escape (Tuekprakhon et al., 2022).

### SARS-CoV-2 variants and molecular detection

For detection of SARS-CoV-2 using the real time-polymerase chain reaction (RT-PCR), multiple genes are targeted in a diagnostic assay (Thakur et al., 2022). This is to ensure specificity and sensitivity of SARS-CoV-2 detection and minimize false positivity. Typical viral genes that are used for such diagnostic purposes include S, N (containing N1 and N2 regions of the N gene), E, nsp12, nsp14, ORF1ab *etc.* (Wang et al., 2020). Particularly, targeting the spike gene ensures specificity as it has unique nucleotide sequences to SARS-CoV-2 and hence minimizes cross-reactivity which would otherwise occur in the presence of other CoVs (Thakur et al., 2022). Given the observed frequent mutation on the S gene, commercial kits and probe sets must be regularly validated to detect new variants and avoid false-negative results (Wang et al., 2020; Thakur et al., 2022). Consistent with this, the S gene target failure has been reported in Alpha and Omicron variants (Wang et al., 2020; Thakur et al., 2022). As a result, a qPCR test that utilize S-gene target failure became useful for rapid detection of the Omicron variant from the Delta variant (Wolter et al., 2022). Conversely, BA.2 lacks the characteristic S-gene target failure, making Delta and BA.2 similar and thus difficult to distinguish the two variants (Rahimi and Talebi Bezmin Abadi, 2022). Owing to the possibility of overlooking BA.2 subvariant (dubbed stealth omicron), S-gene target failure alone may not be sufficient for monitoring the spread of Omicron (Rahimi and Talebi Bezmin Abadi, 2022). Alternatively, BA.2 can be separated from other variants through sequencing or designing primers targeting specific mutations (Rahimi and Talebi Bezmin Abadi, 2022). Of note, although other genes such as N and RdRp (nsp12) are less prone to mutations, any mutation in the primer binding region can reduce assay sensitivity and result in false negative report (Rahman et al., 2021; Wang et al., 2020). For instance, mutations within the N-gene that could affect the sensitivity of RT-PCR tests has been described (Rahman et al., 2021; Wang et al., 2020). Similarly, SNP Q289H which occurred in the N-gene impacted forward primer binding and markedly reduced RT-PCR assay sensitivity (Vanaerschot et al., 2020). Particularly, as omicron variants harbor nine-nucleotide deletions in the N-gene, spanning positions 28,370–28362, single N gene detection-based RT-PCR test assays are expected to produce false negative results (Thakur et al., 2022; Vanaerschot et al., 2020; Wang et al., 2020).

As noted above, the challenge caused by VOCs is clear and there is an urgent need to conduct genetic epidemiological surveys to manage the ongoing pandemic and for better patient care. In this regard, the best approach would be whole genome sequencing (WGS). However, WGS is expensive and time consuming to run on a large scale (Kanjilal and Tang, 2022; Wang et al., 2021d). Alternatively, a rapid and reliable multiplex PCR platform that utilizes a set of four mutation specific PCR based assay has been developed (Wang et al., 2021e). These assays can detect all three RBD spike mutations, including N501Y, E484K, H69-V70del and L450R (Wang et al., 2021e). A similar approach is extended to identify the early circulating SARS-CoV-2 Omicron variant by targeting an Omicron specific Spike (S) insertion-deletion mutation (indel_211–214) observed in B1.1.529/BA.1 lineage and subvariants (Sibai et al., 2022). Similarly, multiplex platforms that can detect five VOC and three variants of interest (VOI) has been developed by screening for spike protein deletions 69 to 70, and 242 to 244 (S-D242-244) as well as S-N501Y, S-E484K, and S-L452R mutations in clinical samples known to be positive for SARS-CoV-2 during the early 2021 (Wang et al., 2021d). Of interest, these multiplex RT-PCR results have 100% concordance with the strains identified by WGS, corroborating their accuracy (Wang et al., 2021d). Understandably, these multiplex RT-PCR based assays may not have high enough throughput to meet the demand of evolving SARS-CoV-2 variants (Boudet et al., 2021), necessitating the development of high throughput screening tests. CoVarScan is a single PCR assay that use 8-plex fragment analysis, detecting eight mutation regions, three recurrently deleted regions (RDR: RDR1, RDR2, and RDR3-4), three RBD mutations (N501Y, E484K, and L452R), and two ORFs (ORF1A and ORF8) (Clark et al., 2022). Thus, CoVarScan has a merit of identifying almost all VOCs, VOIs and capable of detecting the newly emerging SARS-CoV-2 variants (Aoki et al., 2022; Clark et al., 2022), making it a robust detection strategy to efficiently triage a subset of positive samples for confirmation and negative samples for identification using the time consuming and costly whole genome sequencing (WGS).

### Interactions of SARS-CoV-2 with the host and protease required

**FIGURE 6:**
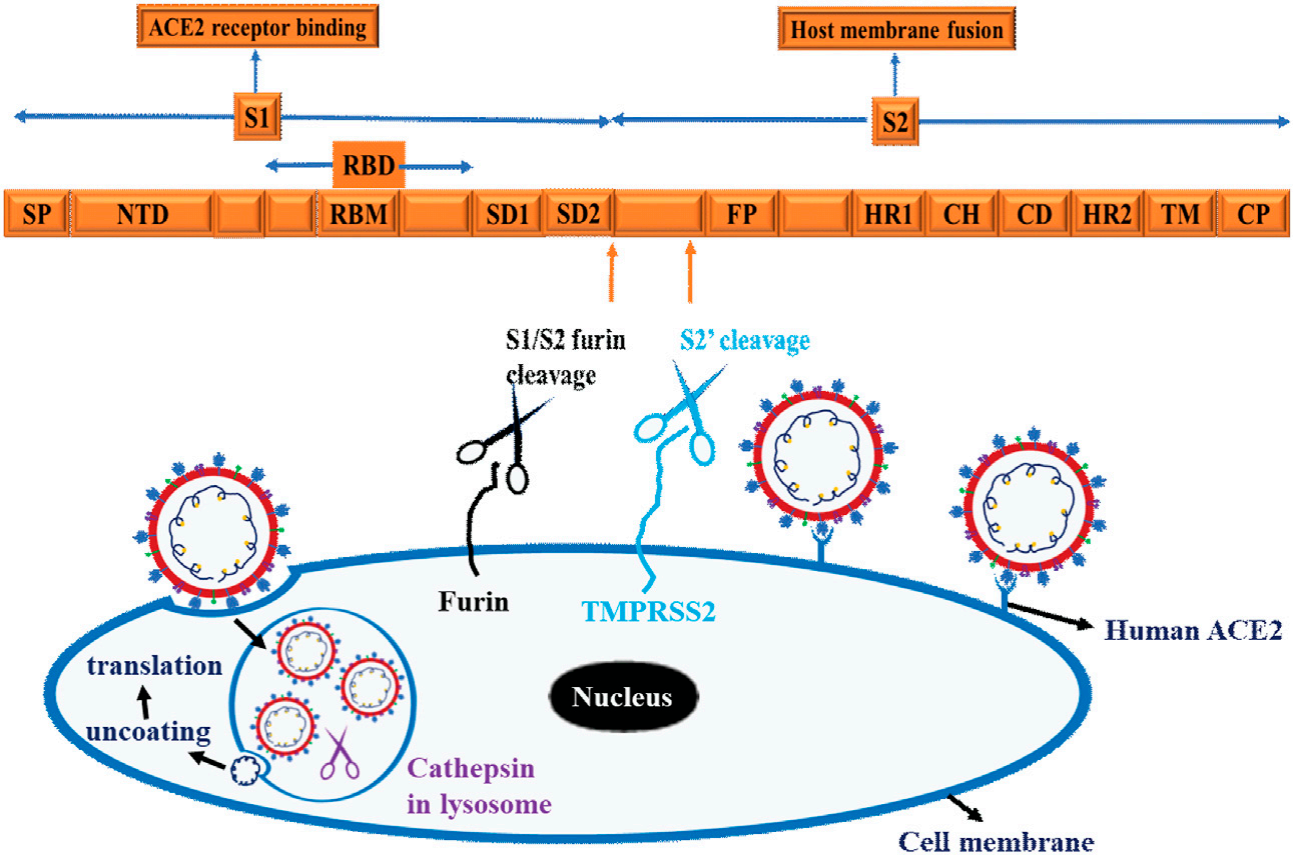
Schematic representation of SARS-CoV-2 and Host cell interaction. The Scheme depicts spike protein, cell binding, endocytosis, viral uncoating, and translation. Three proteases are implicated in the proteolysis of SARS-CoV-2 spike protein, namely Furin, a proprotein convertases, TMPRSS2 (transmembrane protease serine 2), a cell surface protease and cathepsin, a lysosomal protease. These schematics are based on information extracted from various studies (Koppisetti et al., 2021; Naqvi et al., 2020; Zhang et al., 2021) and (Liu et al., 2021).

To initiate successful entry, proteolysis is a fundamental machinery that SARS-CoV-2 uses to deploy fusion peptide for insertion into membrane and gain access into the host cell (Koppisetti et al., 2021). To this end, SARS-CoV-2 binds with the host cell receptor ACE2, which paves the way for the spike protein to undergo proteolysis during infection processes (Naqvi et al., 2020). In this regard, the internal fusion peptide needs to be exposed *via* conformational change (Zhang et al., 2021). These actions ultimately promote plasma membrane fusion (endocytosis), allowing the virus to enter and release its viral RNA into the cytoplasm and for translation to ensue in the host cells (Koppisetti et al., 2021; Naqvi et al., 2020; Zhang et al., 2021) and Figure 6. As shown in Figure 6, three proteases are implicated in the proteolysis of SARS-CoV-2 spike protein, namely furin, a proprotein convertase, TMPRSS2 (transmembrane protease serine 2), a cell surface protease, and cathepsin, a lysosomal protease (Liu et al., 2021). Functionally, furin is a type 1 transmembrane protein that detaches S1 from S2 domain by cleaving the PRRA, a multibasic site at the S1/S2 boundary (Liu et al., 2021). On the other hand, TMPRSS2 is a type 2 transmembrane protein that facilitates membrane fusion by cleaving the S2 site and exposing the internal fusion peptide (Liu et al., 2021; Naqvi et al., 2020). The lysosomal localized cathepsin promote fusion of viral envelope with endosomal membrane by inducing proteolysis after endocytosis of the virion (Liu et al., 2021; Naqvi et al., 2020) and (Figure 6).

### Role of SARS-CoV-2 moiety as PAMPs and TLR as PRRs

Notably, the severity and lethality of SARS-CoV-2 infection is associated with cytokines storm, where SARS-CoV-2 induced hyperactivity of the immune system inflicts damage to the implicated tissue or organs (Choudhury and Mukherjee, 2020; Choudhury et al., 2021a; Patra et al., 2021). Mechanistically, the host innate immune system induces inflammatory responses and eliminates pathogens during infection by recognizing pathogen associated molecular patterns (PAMPs) *via* pattern recognition receptors (PRRs) (Choudhury and Mukherjee, 2020; Choudhury et al., 2021a; Patra et al., 2021). A notable example of PRR is the toll like receptor (TLR) among others (Choudhury and Mukherjee, 2020; Choudhury et al., 2021a; Patra et al., 2021). These SARS-CoV-2 sensing, and inflammatory cytokines production are facilitated by the activation of the MyD88-dependent proinflammatory cytokine production (Kurt-Jones et al., 2000; Zheng et al., 2021). Myd88 is a TLR adapter protein that is required to produce inflammatory cytokines such as TNF-α and IL-6 following β-coronavirus infection (Zheng et al., 2021). In this context, several recent works document the role of various moieties of the SARS-CoV-2 as ligand for the TLR, a PRR. For instance, molecular docking studies have demonstrated significant binding of SARS-CoV-2 native spike protein to TLR1, TLR4, and TLR6 with TLR4 displaying the strongest protein‐protein interaction with spike protein (Choudhury and Mukherjee, 2020). Interestingly, recent study also documented another SARS-CoV-2 component (envelope protein) as a ligand for TLR2 at the extracellular level (Zheng et al., 2021). In this report, E protein induced inflammation comparable with PAMP3 without the need for viral entry (Zheng et al., 2021). Further, a recent *in silico* study documented the role of intracellular TLRs in SARS-CoV-2 mRNA sensing, where TLR3 binds NSP10, TLR7 binds E protein, and TLR9 binds S2 mRNA, suggesting NSP10, E protein and S2 as possible virus‐associated molecular patterns (PAMPs) (Choudhury et al., 2021a). These observations inform the possibility of targeting SARS-CoV-2 moiety (PAMPs) and TLR (PRR) interaction for therapeutic purposes.

### Host immunity, immunomodulation, vaccine, chemotherapy or combinatorial approaches against emergent SARS-CoV-2 variants

As noted above, the spike glycoprotein is the hot spot for genetic alterations, making it an interesting region of the SARS-CoV-2 genome for research and monitoring purposes. Consequently, genomic surveillance of SARS-CoV-2 variants has largely focused on mutations in the spike glycoprotein for two main reasons (Lauring and Hodcroft, 2021; Lopez Bernal et al., 2021). Firstly, S protein mediates attachment to the host cells ACE2 receptor and secondly, it is a major target of neutralizing antibodies produced by host immune responses either following infection or by vaccination (Lauring and Hodcroft, 2021; Lopez Bernal et al., 2021). Given the potential of these genetic alterations to compromise vaccine effectiveness and escape from host antibodies (immune evasion), the intense interest in mutations occurring in the spike glycoprotein and other sites (Wang et al., 2021c; Resende et al., 2021) is a feasible research strategy. The current Omicron variants and its sub-lineages are good examples (Figure 4). Thus, defining these genetic alterations, and their potential influence on vaccine effectiveness, will require large-scale monitoring of SARS-CoV-2 evolution and host immunity. The rise of more transmissible variants like Delta, currently Omicron and its divergent sub-lineages reinforce the need for further SARS-CoV-2 genomic surveillance and alternative therapeutic approaches. As discussed above, cognizant of spike glycoprotein as main arsenals of SARS-CoV-2 infection, its role as a ligand for human TLR4, warrants further therapeutic avenues targeting TLR4. Consistent with this notion, next we attempt to review adjunct therapeutic options.

### Targeting PAMPs/PRRs (SARS-CoV-2 host interaction) with adjunct combinatorial therapy

Cognizant of TLR as the main innate immune receptor recognizing pathogenic ligands and inducing proinflammatory responses, targeting this pathway for therapeutic purpose holds great promise in an effort geared towards mitigation of COVID-19 inflicted immunopathological manifestation (Choudhury and Mukherjee, 2020; Choudhury et al., 2021a; Patra et al., 2021). Thus, in addition to drugs and/or vaccines in current development, understanding the mechanism of inflammatory cytokine production in the host *via* β-coronavirus sensing immunological arsenals such as TLR, opens new approach for adjunct therapeutic strategies to counter the severe illness due to COVID-19 (Choudhury and Mukherjee, 2020; Choudhury et al., 2021a; Patra et al., 2021). In this context, the development of a multi-epitope multi-target chimeric vaccine (named AbhiSCoVac) that stably interact with the TLRs and MHC receptors has been reported using the in slilico based biocomputational approach (Choudhury et al., 2022). Since AbhiSCoVac is hypothesized to work against all pathogenic coronaviruses, it can be a plausible therapeutic approach for managing emerging SARS-CoV-2 variants (Choudhury et al., 2022). Similar approach could be extended for the heart as cardiac injury is another hallmark of covid lethality. Thus, targeting TLR hold great promise for patients inflicted by COVID-19 induced cardiac injury (Choudhury and Mukherjee, 2021). In another context, owing to reduced efficacy of single monoclonal antibodies (MABs) against emerging variants, the need for chimeric antibodies that can target mutant variant and thus display improved efficacy over single MAB has been reported (Das et al., 2021). Importantly, combinatorial therapy that entails chemotherapy and immunotherapy as curative therapeutic remedies needs further investigation (Choudhury et al., 2021b). Thus, immunotherapy such as vaccine that offers preventive role and restore immune homeostasis and, chemotherapy that could potentially thwart host virus interactions, inhibit viral entry, proliferation, and dampen damaging inflammatory environment in COVID-19 patients (Choudhury et al., 2021b) are viable options to explore.

## Concluding remark

Understanding the genome organization of SARS-CoV-2 is indispensable as it familiarizes clinical laboratorians (molecular diagnostics), pathologists (molecular pathology), epidemiologists (molecular epidemiology) and researchers with the products of the viral genome, (proteins enzymes, antigens, and antibodies). As depicted in Figure 1, two-thirds of SARS-CoV-2 genomes encode for viral replicase protein (largest gene coding for ORF1); and the remaining one-third encode for four structural proteins including the S, E, M, and N proteins as well as accessary protein which are embedded within the structural proteins. In this review, we have attempted to provide insight on the genome organization and protein products focusing on the SARS-CoV-2. We have provided useful information regarding the implication of genome structure for understanding emerging variants, such as Alpha, Beta, Delta Gamma (previously circulating VOCs) and Omicron variants (current VOC) and its divergent subvariants (BA.1, BA.2 and BA.3, BA.2.12.1, BA.4 and BA.5) with BA.5 subvariants on the path to become the dominant variant, and is thus being monitored along with others.

Thus, defining these genetic alterations, and their potential influence on detection and therapy/vaccine effectiveness will require large-scale monitoring of SARS-CoV-2 evolution and host immunity. Further, the rise of more transmissible variants like Omicron and its divergent sub-variants reinforce the need for further SARS-CoV-2 genomic surveillance and alternative detection and therapeutic approaches. Thus, future endeavors will focus on the following viable options:

1) The development of rapid and less costly detection tools such as multiplex RT-PCR based assays that has a merit of identifying all VOCs, VOIs and detecting the newly emerging SARS-CoV-2 variants is an important viable option. Strategically, the robustness of such affordable detection tools helps to efficiently triage a subset of positive variants for confirmation and negative samples for further identification using the expensive and time-consuming whole genome sequencing (WGS).
2) The development of combinatorial therapy that entails immunotherapy such as vaccine that offers preventive role and restore immune homeostasis and, chemotherapy that could potentially thwart host virus interactions, inhibit viral entry, proliferation, and dampen damaging inflammatory environment in COVID-19 patients are viable options to explore.

Taken together, information extracted from the genomic structure of SARS-CoV-2 is of paramount importance in variant identification, genomic surveillance, assessment of mechanism of transmission, virulence, and development of specific vaccines tailored to emergent variants, design of chimeric antibodies, and antiviral drugs or combinational therapies.
